# Survey of centers performing cardiovascular magnetic resonance in pediatric and congenital heart disease: a report of the Society for Cardiovascular Magnetic Resonance

**DOI:** 10.1186/s12968-021-00830-4

**Published:** 2022-02-03

**Authors:** Sujatha Buddhe, Brian D. Soriano, Andrew J. Powell

**Affiliations:** 1 Division of Cardiology, Department of Pediatrics, University of Washington School of Medicine and Seattle Children’s Hospital, Seattle, WA USA; 2 Department of Cardiology, Department of Pediatrics, Boston Children’s Hospital, Harvard Medical School, Boston, MA USA

**Keywords:** Cardiovascular magnetic resonance imaging, Pediatric heart disease, Congenital heart disease, Survey

## Abstract

**Background:**

There are few data on practice patterns and trends for cardiovascular magnetic resonance (CMR) in pediatric and congenital heart disease. The Society for Cardiovascular Magnetic Resonance (SCMR) sought to address this deficiency by performing an international survey of CMR centers.

**Methods:**

Surveys consisting of 31 (2014) and 33 (2018) items were designed to collect data on the use of CMR for the evaluation of pediatric and congenital heart disease patients. They were sent to all SCMR members in 2014 and 2018. One response per center was collected.

**Results:**

There were 93 centers that responded in 2014 and 83 in 2018. The results that follow show data from 2014 and 2018 separated by a dash. The median annual number of pediatric/congenital CMR cases per center was 183–209. The median number of scanners for CMR was 2–2 (range, 1–8) with 58–63% using only 1.5T scanners and 4–4% using only 3T scanners. The mean number of attending/staff reading CMRs was 3.7–2.6; among them, 52–61% were pediatric or adult cardiologists and 47–38% were pediatric or adult radiologists. The median annual case volume per attending was 54–86. The median number of technologists per center doing CMRs was 4–5. The median scanner time allocated for a non–sedated examination was 75–75 min (range, 45–120). Among the 21 centers responding to both surveys, the mean annual case volume increased from 320 in 2014 to 445 in 2018; 17 (81%) of the centers had an increase in annual case volume. For this subgroup, the median attending/staff per center was 4 in both 2014 and 2018. The median scanner time allotted per study was unchanged at 90 min. The mean time for an attending/staff physician to perform a typical CMR examination including reporting was 143–141 min.

**Conclusion:**

These survey data provide a novel comprehensive view of CMR practice in pediatric and congenital heart disease. This information is useful for internal benchmarking, resource allocation, addressing practice variation, quality improvement initiatives, and identifying unmet needs.

**Supplementary Information:**

The online version contains supplementary material available at 10.1186/s12968-021-00830-4.

## Background

The use of cardiovascular magnetic resonance (CMR) for the evaluation of pediatric and congenital heart disease continues to grow and evolve. The numerous indications for this patient group have been delineated by various professional societies [1–3]. These include a wide variety of congenital and acquired conditions as well as scenarios such as initial diagnosis, pre-interventional planning, and serial follow-up. In many centers, CMR is fully integrated into clinical care alongside other traditional imaging modalities such as echocardiography and invasive cardiac catheterization. Despite this clear maturation, there are few multicenter data that describe CMR practice patterns and temporal trends in pediatric and congenital heart disease. This may limit a center’s ability to review their practices, evaluate their program, and identify unmet needs. Professional societies such as the American Society of Echocardiography and the American Academy of Pediatrics have periodically surveyed centers regarding several practice components to address resource allocation and provide benchmarks [4–11] .

Under the auspices of the Society for Cardiovascular Magnetic Resonance (SCMR), we performed an international survey of CMR centers/programs caring for patients with pediatric or congenital heart disease. The survey was performed in 2014 and again in 2018 to assess temporal trends.

## Methods

A survey consisting of 31 items in 2014 and 33 items in 2018 was designed with input from members of the SCMR Pediatric and Congenital Heart Disease Section Steering Committee. All items from the 2014 survey were included in the 2018 survey. The surveys were sent to all SCMR members by email in 2014 and then in 2018 using *SurveyMonkey* (San Mateo, California, USA) and posted on the SCMR website and Twitter account. One response per center was requested and included. The 2018 survey is shown as Additional file 1. The items included description of the program/center; CMR patient volume and age distribution; center echocardiogram and cardiac surgical volumes; CMR scanner type; use of sedation, anesthesia, and breath-holding; physician number, specialty, and co-read practices; technologist number; center accreditation status; physician presence during examinations; CMR scanning days per week; scanner time allotted for typical examinations; personnel performing post-processing; image analysis software used for chamber volume and blood flow; average time spent per examination by physicians; gadolinium-based contrast agents used; sequences performed and reported in clinical reports; and interest in participating in future surveys.

Data are reported as mean and standard deviation for normally distributed continuous variables, median and range for non-normally distributed continuous variables, and number and percentage for categorical variables. A subgroup analysis was performed for centers responding to both surveys. All statistical analyses were performed using SPSS (version 19.0, Statistical Package for the Social Sciences, International Business Machines, Inc., Armonk, New York, USA).

## Results

A total of 93 centers responded in 2014 and 83 in 2018, including 21 centers that responded to both surveys. In the results that follow, values from 2014 to 2018 are separated by a dash. Nearly all centers (97–100%) responded that they were interested in participating in future surveys.

### Center characteristics

**Fig. 1:**
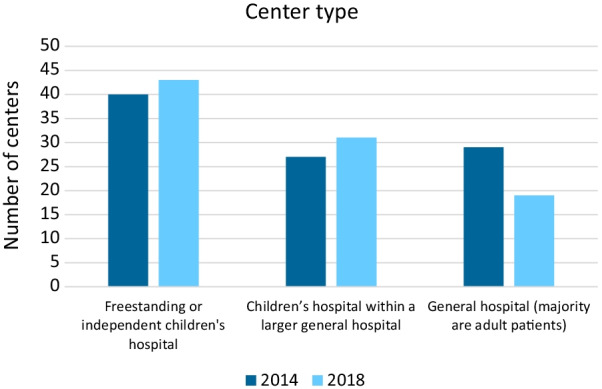
Center/program type

Table 1 shows the countries where the responding centers were located. The majority (62–68%) of responders were located in the United States. Among all centers, 40–43% identified themselves as a free-standing or independent children’s hospital; 27–31% as a children’s hospital within a larger general hospital, and 27–19% as a general hospital where the majority of patients are adults (Fig. 1). Among centers in the United States, only American College of Radiology accreditation was obtained by 68–68%, only Intersocietal Accreditation Commission – Magnetic Resonance Imaging accreditation by 8–6%, and both accreditations by 9–13%.

**Table 1:** Survey centers by country

Country	2014		2018	
	N	%	N	%
United States	57	62.0	49	68.0
United Kingdom	9	10.0	1	1.4
Brazil	5	6.0	2	2.8
Canada	4	4.0	2	2.8
Australia	2	2.0	1	1.4
Colombia	2	2.0	0	0
Germany	2	2.0	2	2.8
Italy	2	2.0	1	1.4
Belgium	1	1.0	0	0
Finland	1	1.0	0	0
Hong Kong SAR	1	1.0	1	1
Mexico	1	1.0	0	0
Poland	1	1.0	0	0
Spain	1	1.0	0	0
Sweden	1	1.0	0	0
Switzerland	1	1.0	0	0
Thailand	1	1.0	0	0
The Netherlands	1	1.0	0	0
Argentina	0	0	1	1.4
Czech Republic	0	0	1	1.4
Egypt	0	0	1	1.4
France	0	0	1	1.4
Greece	0	0	1	1.4
Hungary	0	0	1	1.4
Japan	0	0	1	1.4
Kuwait	0	0	1	1.4
Lebanon	0	0	1	1.4
New Zealand	0	0	1	1.4
Norway	0	0	1	1.4
Romania	0	0	1	1.4
Saudi Arabia	0	0	1	1.4

### Patient volume and age

**Fig. 2:**
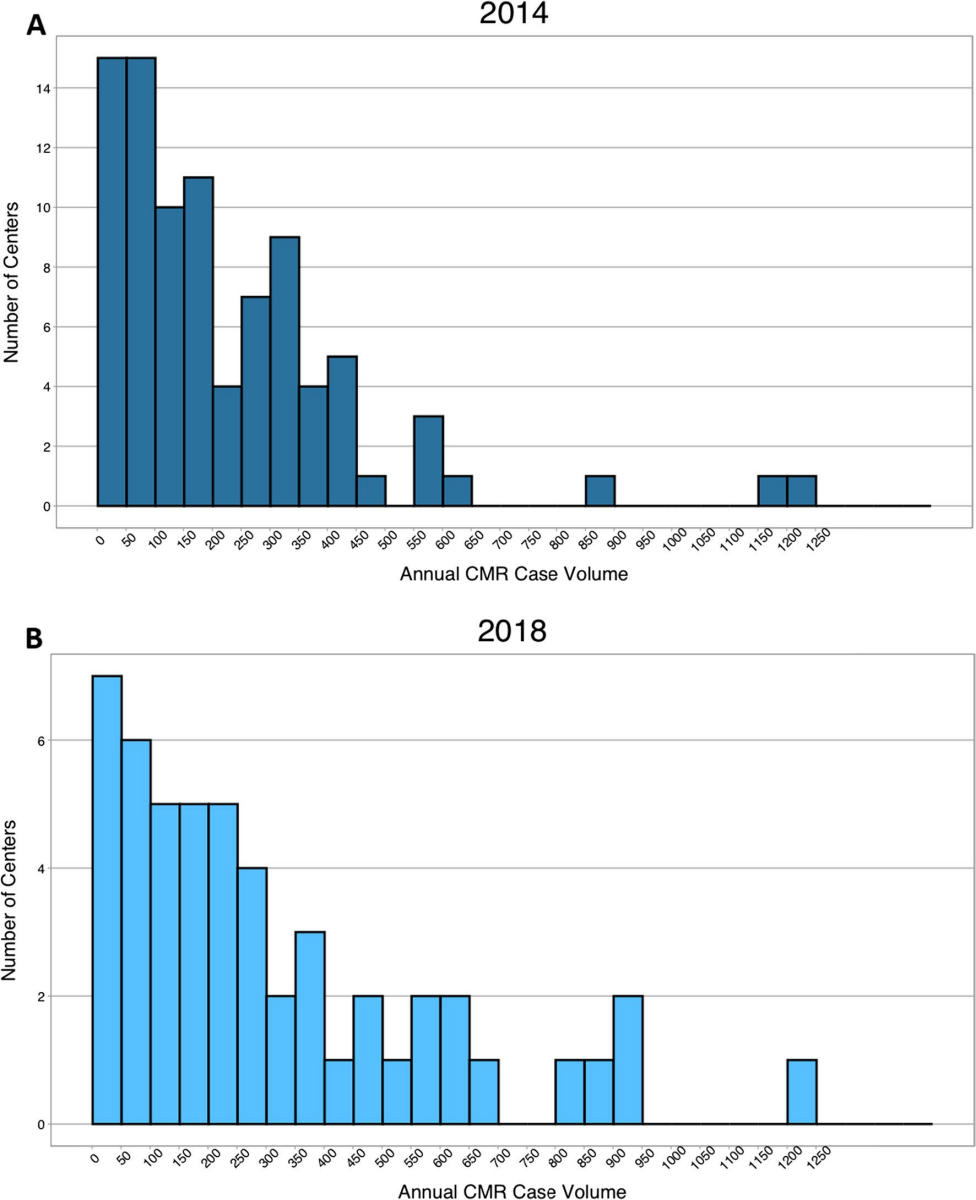
Annual pediatric and congenital heart disease patient CMR case volume per center in 2014 and 2018

The annual pediatric and congenital heart disease patient CMR case volume per center is shown in Fig. 2; the median was 183–209 cases. The annual pediatric and congenital heart disease patient echocardiogram case volume per center was <5000 in 32–28%, 5000 to 10,000 in 32–28%, 10,000 to 15,000 in 21–13%, and >15,000 in 15–28%. The annual pediatric and congenital heart disease surgery case volume per center was none in 13–11%, <250 in 30–39%, 250 to 500 in 38–26%, and >500 in 19–24%. Patients of all ages underwent CMR in 72–77% of the centers, only patients older than one year were scanned in 15–17% of the centers, only patients older than 18 years were scanned in 6–3% of the centers, and only patients younger than 18 years were scanned in 7–3% of the centers.

### Imaging technique

The median number of scanners used for CMR per center was 2–2 (range, 1–8) and 66–78% used more than one scanner. The scanner manufacturer was Siemens in 51–49%, Philips in 27–27%, General Electric in 22–22%, and Toshiba in 0–2%. Among centers with more than one CMR scanner, 79–75% had a single scanner manufacturer. Regarding the CMR scanner field strength, only a 1.5T scanner was used in 58–63% of the centers and only a 3T scanner was used in 4–4% of the centers; the remainder used both field strengths.

Sedation and/or anesthesia for some CMR cases was used in 84–86% of the centers. Among these centers, when using sedation and/or anesthesia, general anesthesia (defined as the use of endotracheal intubation or a laryngeal mask airway) was the predominant approach (>90% of cases) in 64–50% of centers, sedation without endotracheal intubation or a laryngeal mask airway was the predominant approach (>90% of cases) in 23–11% of centers, and a varied approach with either sedation or general anesthesia was used in the remaining 13–39% of the centers. For cases using general anesthesia, ventilation was intermittently suspended to decrease respiratory motion artifact in ≥50% of the cases at 64–52% of centers, ventilation was intermittently suspended to decrease respiratory motion artifact in <50% of the cases at 16–33% of the centers, and ventilation was never suspended at 20–15% of centers.

Only the 2018 survey queried the type of gadolinium-based contrast agent (GBCA). Only a macrocyclic GBCA was used in 79% of centers, only a linear GBCA was used in 12% of centers, and both types of GBCAs were used in 9% of centers. In 2014, 27% of centers reported the use of gadofosveset (trade name Ablavar and Vasovist, Lantheus Medical Imaging, North Billerica, Massachusetts, USA). In 2018, this agent was no longer commercially available. In 2018, 17% of all centers and 29% of centers in the United States used ferumoxytol (trade name Feraheme, AMAG Pharmaceuticals, Waltham, Massachusetts, USA) as a contrast agent. Among the centers that performed CMR examinations in patients <30 days of age, GBCAs were used in patients <30 days of age in 62–71%.

In the 2018 survey, 38 centers responded to questions on the use of newer techniques. Among them, four-dimensional (4D) flow sequences were acquired by 55% of the centers and 4D flow data were included in the clinical report in 18% of the centers. T1 mapping sequences were performed by 97% of the centers and data from T1 mapping was included in the clinical report in 66% of the centers. Three-dimensional black-blood sequences were performed in 24% of the centers, feature tracking in 55% of the centers, tissue phase mapping in 18% of the centers, and compressed sensing in 32% of the centers.

### Staff

**Fig. 3:**
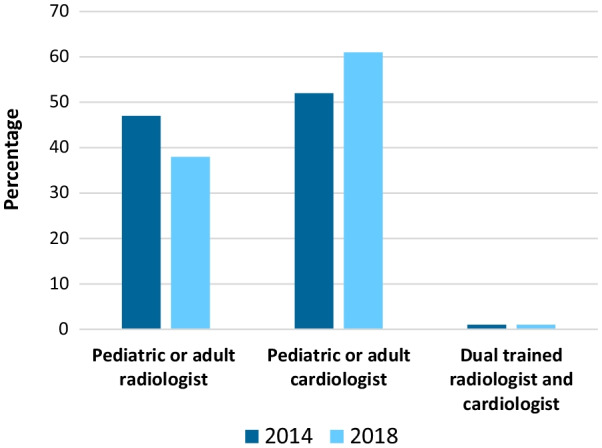
Specialty of attending/staff physicians reporting CMR

The mean number of attending/staff who report pediatric/congenital CMR examinations per center was 3.7–2.6. The median case volume per attending/staff physician per year was 54–86. Among the attending/staff physicians reporting pediatric/congenital CMR examinations, 52–61% were pediatric or adult cardiologists, 47–38% were pediatric or adult radiologists, and 1–1% were dual trained radiologist and cardiologists (Fig. 3). A more detailed breakdown by specialty training of this group is shown in Table 2. The official signer of the CMR reports by specialty is presented in Table 3. Physician training in pediatric/congenital CMR was provided at 41–43% of the centers. The median number of technologists per center performing pediatric/congenital CMR examinations was 4–5. The median case volume per technologist per year was 45–43.

**Table 2:** Specialty training of attending/staff reporting pediatric/congenital CMR examinations

Specialty training	2014 (%)	2018 (%)
Pediatric cardiology	28	45
Pediatric radiology	20	19
Adult cardiology	21	12
Adult radiology	19	15
Adult and pediatric cardiology	3	4
Adult and pediatric radiology	8	4
Radiology and cardiology	1	1

**Table 3:** Official signer of CMR reports by specialty

Signer	2014 (%)	2018 (%)
Only cardiologists officially sign reports	30	34
Only radiologists officially sign reports	27	4.5
Only one physician signs each report, and this may be either a cardiologist or radiologist (1 report per patient)	21	23
Both radiologists and cardiologists officially sign all reports (2 physicians per report)	12	25
Radiologists and cardiologists sign some studies jointly and some by themselves	10	9
A radiologist and a cardiologist each sign separate reports on the same patient (2 reports per patient)	0	4.5

### Workflow and resources

CMR examinations were performed 1 day per week at 19–30% of centers, 2–4 days per week at 45–43% of centers, and ≥5 days per week at 29–27% of centers. The median scanner time allocated for a non-sedated examination was 75–75 min (range, 45–120). Physicians were typically present in the same room as the technologist for >60% of the examination time in 91–86% of centers. The mean time for an attending/staff physician to perform and report a typical CMR examination was 126–130 min.

The initial contour drawing for analysis of ventricular volume and flow measurements was performed by an attending/staff physician only at 37–32% of centers, a physician trainee only in 9–5%, a technician only in 5–7%, a technologist only in 5–7%, and a combination of these in 44–24%. An in-house post-processing team (e.g., 3D laboratory) or third-party company was used in 25% of centers in 2018; it was not a response option in 2014. For ventricular volume calculation, the software vendor was Siemens (Siemens Healthineers, Erlangen, Germany) at 31–9%, Medis (Medis Medical Imaging, Leiden, The Netherlands) at 25–26%, Philips (Philips Healthcare, Best, the Netherlands) at 20–5%, General Electric (General Electric Healthcare, Chicago, IL, USA) at 11–7%, cvi42 (Circle Cardiovascular Imaging, Calgary, Alberta, Canada) at 9–34%, Osirix (Osirix Foundation, Geneva, Switzerland) at 1–2%, Segment CMR (Medviso, Lund, Sweden) at 1–2%, Heart Imaging Technologies (Durham, North Carolina, USA) at 0–5%, TeraRecon (Acton, Massachusetts, USA) at 0–5%, Arterys (San Francisco, CA, USA) at 0–4% and other at 2–1%. For blood flow calculation, the software vendor was Siemens at 34–9%, Philips at 23–7%, Medis at 20–25%, General Electric at 12–5%, cvi42 at 8–34%, Segment CMR at 1–4%, Heart Imaging Technologies at 0–7%, TeraRecon at 0–5%, and other at 2–4%.

### Centers responding to both surveys

A total of 21 centers responded to both surveys. The mean case volume per year increased from 320 in 2014 to 445 in 2018; 17 (81%) of the centers had an increase in case volume per year. The median number of attending/staff physicians reporting CMR examinations per center was 4 in both 2014 and 2018. Among the attending/staff physicians reporting CMR examinations, 62–65% were pediatric or adult cardiologists and 38–35% were pediatric or adult radiologists. The median scanner time allocated for a non-sedated examination was 90 min in both 2014 and 2018. The mean time for an attending/staff physician to perform a typical CMR examination including reporting was 143 min in 2014 and 141 min in 2018.

## Discussion

This report is the first comprehensive international survey of CMR centers/programs caring for patients with pediatric or congenital heart disease. The survey was voluntary and distributed in 2014 and then again in 2018. Overall, it demonstrated significant variation in program size, case volume, and some practices.

In both survey years, approximately two-thirds of the responding centers were in the United States. This likely overrepresents the United States compared to other countries throughout the world but is reflective of the SCMR membership. Most centers were independent children’s hospital, followed by children’s hospital within a larger general hospital and then a general hospital where majority of patients are adults. It is unclear how well this distribution reflects all CMR centers worldwide caring for patients with pediatric or congenital heart disease. It seems possible that independent children’s hospitals may be more common in the United States than in other countries as a whole, thereby skewing the results. Nearly all centers in the survey also performed surgery on pediatric and congenital heart disease patients. This high proportion raises the possibility that smaller centers and dedicated imaging centers may be underrepresented.

This survey yielded interesting information about CMR imaging techniques in pediatric and congenital heart disease patients. With regard to scanner field strength, the majority of centers used only a 1.5T scanner while just 4% used only a 3T scanner; the remainder used both a 1.5T and a 3T scanner. The more frequent use of 1.5T scanners may be a reflection of them being more common or accessible at the survey centers. However, the results, particularly the few centers relying only on a 3T scanner, may also indicate a preference for the lower field strength even when both are available. We speculate that this could be related to differences in image quality with banding artifacts from off-resonance effects being more common at 3T. Moreover, metallic implanted devices (e.g., stents, vascular occlusion coils, and prosthetic valves) are relatively common in patients with congenital heart disease and produce more image artifact at higher field strength. Although some cardiac applications such as coronary imaging, myocardial perfusion imaging, and myocardial tagging are likely superior at 3T, the need for these in pediatric and congenital heart disease patients may be less prominent than that in patients with adult-onset conditions. The use of sedation or anesthesia for some CMR examinations was common among the responding centers. Some centers preferred to use general anesthesia over sedation while others had a mixed approach. This variation is likely in part because general anesthesia requires specialized personnel, equipment, and resources [12–15] that are not universally available. Lastly, it is notable that in the 2018 survey, macrocyclic GBCAs were more commonly used than linear GBCAs. This likely stems from studies that show a higher likelihood of cerebral gadolinium deposition with linear GBCAs compared to macrocyclic GBCAs [16–18]. Nevertheless, the clinical relevance of these depositions has yet to be determined [19].

This survey demonstrates that CMR in patients with pediatric or congenital heart disease is performed by physicians with a variety of subspecialty certifications; these included adult and pediatric radiology, and adult and pediatric cardiology [20–22]. The proportion of these specialists reported in this survey may not be representative of the field as a whole because of sampling biases. The median case volume per attending/staff physician per year was 54 in 2014 and increased to 86 in 2018, and per technologist per year was 45 in 2014 and 43 in 2018. In comparison to the volumes seen in other cardiac imaging modalities (e.g., echocardiography), these volumes are relatively small.

Our survey data on workflow support the notion that CMR examinations in patients with pediatric or congenital heart disease are time and resource intensive compared to other types of magnetic resonance examinations. The median scanner time allocated for a non-sedated examination was approximately 75 min. The mean time for an attending/staff physician to perform a typical CMR examination including reporting was approximately 130 min. Physicians were typically present in the same room as the technologist for >60% of the examination time at nearly all centers. This information may be useful to ensure that appropriate financial and physician work credit is assigned to this type of examination. Although the explanation for the resource utilization was not explicitly assessed in the survey, CMR examinations in patients with pediatric or congenital heart disease may involve time-consuming coaching to elicit patient cooperation, complex anatomy and unexpected findings such that the imaging protocol needs expert supervision and tailoring in real-time, and multiple relevant clinical questions that add to the examination and reporting time. Of note, centers that responded to both surveys had on-average longer scan slots and interpretation times despite presumably a greater experience and larger volumes than the average in the other groups. While it is unlear if this could be related to adding new sequences, overall this data suggests that experience may not decrease scan times or interpretation times. The survey’s results also highlight the need to develop faster scanning techniques and automated image analysis to maximize efficiency [23–25].

### Limitations

The generalizability of this survey is limited by potential selection bias (detailed above) and response bias. No comprehensive list of CMR centers/programs caring for patients with pediatric or congenital heart disease is available. Beyond goodwill, the only incentive offered to participate was access to the results as soon as they were collated. Another limitation to this survey was that data from centers are self-reported and were not independently validated. Future surveys should aim for more widespread dissemination and consider offering additional incentives to respond.

## Conclusions

This survey data provides a novel comprehensive view of CMR practice in pediatric and congenital heart disease from centers across the globe. The information is useful for internal benchmarking, identifying unmet needs, addressing practice variation, resource allocation, and developing quality improvement initiatives. It also serves as a foundation for future surveys.

## Supplementary Information

**Additional file 1. **Pediatric/congenital CMR survey.

## Data Availability

The datasets during and/or analyzed during the current study are available from the corresponding author on reasonable request.

## Abbreviations

4D: 4 Dimensional
CMR: Cardiovascular magnetic resonance
GBCA: Gadolinium-based contrast agents
SCMR: Society for Cardiovascular Magnetic Resonance

## Declarations

### Peer Review Status

This article is a publication of the Society of Cardiovascular Magnetic Resonance – it has been endorsed by the Society and assessed by the Editor-in-Chief prior to publication.
