# Supplementary material for: Survey of centers performing cardiovascular magnetic resonance in pediatric and congenital heart disease: a report of the Society for Cardiovascular Magnetic Resonance
Source: J Cardiovasc Magn Reson. 2022 Feb 3;24:10. doi: 10.1186/s12968-021-00830-4 (PMC8812017; doi:10.1186/s12968-021-00830-4)
Supplement: Supplementary file 1 — Additional file 1. Pediatric/congenital CMR survey. [file 12968_2021_830_MOESM1_ESM.pdf]

Pediatric/Congenital CMR Survey

Introduction

Aim: To collect and to update information on the practice of cardiovascular magnetic resonance (CMR) in patients with pediatric or congenital heart disease by center/program. This information should be useful to assess practice variation and serve as the foundation for future surveys, quality improvement initiatives, and community activities. This is a repeat assessment, using questions derived from the 2014 survey.

Methods: One self-report survey to be completed by each center. Each center should designate one individual to respond.

Results: Survey results will be distributed to each respondent. Centers will not be identified in the distributed report. Results will be compared between this time frame and the 2014 survey results. Our findings may be published at a later date.

Time: Approximately 20 minutes to complete.

Request: The usefulness of the survey depends entirely on the accuracy of the self-reporting. Please do your best to answer all questions faithfully.

Tip: A PDF of the survey is attached to the email. Please view it first so that you can be prepared to enter the data into the on-line survey.

Deadline: Please complete the survey by October 1, 2018.

Contact: Sujatha Buddhe (sujatha.buddhe@seattlechildrens.org);Brian D Soriano (brian.soriano@seattlechildrens.org); Andrew Powell, MD (andrew.powell@cardio.chboston.org)

Demographics

* 1. Responder:

Name: ____________________

Email Address: ____________________

* 2. CMR program name. If your program images at multiple institutions/locations, list the primary one.

Name: ____________________

Address 1: ____________________

Address 2: ____________________

City/Town: ____________________

State/Province: ____________________

ZIP/Postal Code: ____________________

Country: ____________________

* 3. Which description best fits your program/center?

- ◯ General hospital (majority of patients are adults)
- ◯ Free-standing or independent children's hospital
- ◯ Children's hospital within a larger general hospital
- ◯ Imaging center not affiliated with a hospital
- ◯ Other (please specify)

____

Case volume

A pediatric or congenital CMR examination is defined as one in which the primary goal of the study is to investigate known or suspected pediatric or congenital heart disease. Examinations in which only angiography is performed (e.g., possible vascular ring) should be included.

4. Case volume data should be for calendar year 2017. If you are unable to provide data for this time period, please specify the time period in this field:

5. Total number of pediatric and congenital CMR cases:

All ages

Comment

6. Age-based number of pediatric and congenital CMR cases (total should equal response to question 5 above):

Age <1 year

Age 1-18 years

Age >18 years

Comment

* 7. Do you perform pediatric or congenital heart disease CMR cases with sedation and/or anesthesia? Do not include cases in which only oral anxiolysis medications were used (e.g., for claustrophobia).

- No
- Yes

8. If yes, case volume:

Sedation (no breathing tube or laryngeal mask airway used)

Anesthesia (breathing tube or laryngeal mask airway used)

Comment

9. Echocardiogram case volume for pediatric and congenital heart disease patients over the same time period for all ages:

- ○ None
- ○ 1-5000
- ○ 5001-10,000
- ○ 10,001-15,000
- ○ 15,001-20,000
- ○ >20,000

Exact number if known

[ ]

10. Cardiac surgery (on and off bypass) case volume for pediatric and congenital heart disease patients over the same time period for all ages:

- ○ None
- ○ 1-50
- ○ 51-250
- ○ 251-500
- ○ 501-750
- ○ 751-1000
- ○ >1000

Exact number if known

[ ]

MR scanner equipment

11. MR scanners used for clinical pediatric/congenital cardiac imaging:

	Manufacturer	Field strength	Number of scanners
Scanner type 1			
Scanner type 2			
Scanner type 3			
Scanner type 4			
Scanner type 5			

Other (please specify). If possible please provide actual model number.

Staffing, training, and accreditation

12. Number of attending/staff (non-trainee) physicians who report pediatric/congenital CMR examinations:

Adult radiologists

Pediatric radiologists

Dual-trained adult and pediatric radiologists

Adult cardiologists

Pediatric cardiologists

Dual-trained adult and pediatric cardiologists

Dual-trained radiologist and cardiologists

Other

Comment

13. Number of individual technologists who perform pediatric/congenital CMR examinations:

Technologists

14. Does your center provide physician training designed to provide the skills necessary to independently interpret pediatric/congenital CMR examinations (i.e., level 2 or 3 training and not simply general training to provide a working knowledge of CMR methods and diagnostic utility)?

- Yes
- No

15. For centers in the United States, what is your accreditation status?

- [ ] Accreditted by the American College of Radiology (ACR)
- [ ] Accreditted by the Intersocietal Accreditation Commission (IAC)
- [ ] None
- [ ] Other (please specify)

Workflow

16. Percentage of pediatric/congenital CMR examinations in which a physician is in the same room as the technologist during at least some of the image acquisition:

- ◯ <10% of examinations
- ◯ 10-40% of examinations
- ◯ 41-60% of examinations
- ◯ 61-90% of examinations
- ◯ >90% of examinations

17. When present in the same room as the technologist during an examination, the physician is typically there for:

- ◯ <10% of the examination
- ◯ 10-40% of the examination
- ◯ 41-60% of the examination
- ◯ 61-90% of the examination
- ◯ >90% of the examination

18. For pediatric/congenital CMR reporting, which of the following best describes your center:

- ◯ Only radiologists officially sign reports
- ◯ Only cardiologists officially sign reports
- ◯ Both a radiologist and a cardiologist officially sign all reports (2 physicians per report)
- ◯ A radiologist and a cardiologist each sign separate reports on the same patient (2 reports per patient)
- ◯ Radiologists and cardiologists sign some studies jointly and some by themselves
- ◯ Only one physician signs each report, and this may be either a cardiologist or radiologist (1 report per patient)

Other/comment

19. How many days per week do you ROUTINELY schedule pediatric/congenital CMR examinations?

Comment

20. What is the typical amount of scanner time (in minutes) allotted for a CMR examination without anesthesia or sedation?

Comment

21. If you allot a range of times for a CMR examination without anesthesia or sedation, what is the shortest amount of time?

Comment

22. Who usually performs the initial tracing of ventricular and blood vessel contours for quantitative analysis? Select more than one if there is an even division.

- [ ] MR technologist
- [ ] Physician trainee
- [ ] Medical student
- [ ] Attending/staff physician
- [ ] Technician
- [ ] Third party company
- [ ] In house post-processing team (e.g., 3D lab)

Other (please specify)

23. What software do you usually use for measuring ventricular volume?

- [ ] Arterys
- [ ] Compass (Cardiom)
- [ ] cmr42 (Circle Cardiovascular Imaging)
- [ ] CMRtools (Cardiovascular Imaging Solutions)
- [ ] General Electric workstation
- [ ] Heart Imaging Technologies
- [ ] HeartPro (MedVoxel Systems)
- [ ] NeoSoft
- [ ] Osirix
- [ ] PIE Medical Imaging
- [ ] Philips workstation
- [ ] QMass (Medis)
- [ ] Segment CMR (Medviso)
- [ ] Siemens workstation
- [ ] TeraRecon
- [ ] Custom/non-commercial

Other (please specify)

24. What software do you usually use for measuring blood flow?

- [ ] Arterys
- [ ] Compass (Cardiom)
- [ ] cmr42 (Circle Cardiovascular Imaging)
- [ ] CMRtools (Cardiovascular Imaging Solutions)
- [ ] General Electric workstation
- [ ] Heart Imaging Technologies
- [ ] HeartPro (MedVoxel Systems)
- [ ] NeoSoft
- [ ] Osirix
- [ ] Philips workstation
- [ ] PIE Medical Imaging
- [ ] QFlow (Medis)
- [ ] Segment CMR (Medviso)
- [ ] Siemens workstation
- [ ] TeraRecon
- [ ] Custom/non-commercial

Other (please specify)

25. What is the average number of minutes it takes an attending/staff physician to perform a typical pediatric/congenital CMR examination including supervision, scanning, analysis, and reporting time?

26. Do you administer gadolinium-based contrast agents to newborns (age <30 days)?

- ○ We do not perform MR examinations on patients age <30 days at our center
- ○ Yes
- ○ No

27. What contrast agents are you using currently (select all that apply):

- [ ] Linear gadolinium based contrast agents
- [ ] Macrocyclic gadolinium based contrast agents
- [ ] Feraheme (ferumoxytol)
- [ ] Other (please specify)

28. In approximately what percent of examinations under general anesthesia do you suspend respiration (breath-hold) to minimize respiratory motion artifact?

- ○ >75%
- ○ 50-75%
- ○ 10-50%
- ○ <10%
- ○ Never

Comment

29. Is your institution acquiring/performing any of the relatively new sequences (select all that apply):

- [ ] 4D flow
- [ ] T1 mapping
- [ ] Tissue phase mapping/ Feature tracking
- [ ] 3D black blood imaging
- [ ] Compressed sensing
- [ ] Other (please specify)

30. Is your institution including any of the relatively new sequences in the clinical report (select all that apply):

- [ ] 4D flow
- [ ] T1 mapping
- [ ] Tissue phase mapping/ Feature tracking
- [ ] 3D black blood imaging
- [ ] Compressed sensing
- [ ] Other (please specify)

Conclusion

31. Are you willing to participate in future surveys about pediatric/congenital CMR?

- ○ Yes
- ○ No

32. What questions or topics would you like future surveys to address?

33. Any additional comments on this survey?

Thank you for completing this important survey!
